# Investigating safety profiles of human papillomavirus vaccine across group differences using VAERS data and MedDRA

**DOI:** 10.7717/peerj.7490

**Published:** 2019-08-20

**Authors:** Yuxi Jia, Cong Zhu, Jingcheng Du, Yang Xiang, Yong Chen, Wei Wang, Cui Tao

**Affiliations:** 1 Department of Medical Informatics, School of Public Health, Jilin University, Changchun, Jilin Province, China; 2 School of Biomedical Informatics, University of Texas Health Center at Houston, Houston, TX, USA; 3 Department of Epidemiology, Human Genetics, and Environmental Sciences, School of Public Health, University of Texas Health Science Center at Houston, Houston, TX, USA; 4 Department of Biostatistics and Epidemiology, University of Pennsylvania, Philadelphia, PA, USA

**Keywords:** Adverse events, Clustering analyses, Human papillomavirus vaccine, VAERS, MedDRA, Post market surveillance, Pharmacovigilance, Vaccine safety

## Abstract

**Background:**

The safety of vaccines is a critical factor in maintaining public trust in national vaccination programs. This study aimed to evaluate the safety profiles of human papillomavirus (HPV) vaccines with regard to the distribution of adverse events (AE) across gender and age, and the correlations across various AEs using the Food and Drug Administration/Centers for Disease Control and Prevention Vaccine Adverse Event Reporting System (VAERS).

**Methods:**

For analyses, 27,348 patients aged between 9 and 25 years old with at least one AE reported in VAERS between the year of 2006 and 2017 were included. AEs were summarized into two levels: the lower level preferred term (PT) and higher level system organ classes (SOCs) based on the structure of Medical Dictionary for Regulatory Activities (MedDRA). A series of statistical analyses were applied on both levels of AEs. Zero-truncated Poisson regression and multivariate logistic regression models were first developed to assess the rate and risk of SOCs across age groups and genders. Pairwise Pearson correlation analyses and hierarchical clustering analyses were then conducted to explore the interrelationships and clustering pattern among AEs.

**Results:**

We identified 27,337 unique HPV vaccine reports between 2006 and 2017. Disproportional reporting of AEs was observed across age and gender in 21 SOCs (*p* < 0.05). The correlation analyses found most SOCs demonstrate weak positive correlations except for five pairs which were negatively correlated: skin and subcutaneous tissue disorders + injury poisoning and procedural complications; skin and subcutaneous tissue disorders + nervous system disorders; Skin and subcutaneous tissue disorders + pregnancy, puerperium and perinatal conditions; nervous system disorders + pregnancy, puerperium and perinatal conditions; pregnancy, puerperium and perinatal conditions + general disorders and administration site conditions. Nervous system disorders had the most AEs which contributed to 12,448 (46%) cases. In the further analyses of correlations between PT in nervous system disorders, the three most strongly correlated AEs were psychiatric disorders (*r* = 0.35), gastrointestinal disorders (*r* = 0.215), and musculoskeletal and connective tissue disorders (*r* = 0.261). We observed an inter-SOCs correlation of the PTs among AE pairs by nervous system disorders/psychiatric disorders/gastrointestinal disorders/musculoskeletal and connective tissue disorders.

**Conclusions:**

The analyses revealed a different distribution pattern of AEs across gender and age subgroups in 21 SOC level AEs. Correlation analyses and hierarchical clustering analyses further revealed several correlated patterns across various AEs. However, findings from this study should be interpreted with caution. Further clinical studies are needed to understand the heterogeneity of AEs reporting across subgroups and the biological pathways among the statistically correlated AEs.

## Background

Based on statistics collected by the Centers for Disease Control and Prevention (CDC) (2017b), approximately 33,700 incidences of cancer per year are associated with human papillomavirus (HPV) infection. HPV is considered a leading cause of cervical cancers (Walboomers et al., 1999), respiratory papilloma, as well as of several types of cancers of the vagina, vulva, penis, anus, rectum and oropharynx (Hoory et al., 2008; Lele et al., 2002). To prevent HPV infection and HPV related cancers (Centers for Disease Control and Prevention (CDC), 2016a; Cutts et al., 2007; Sundaram et al., 2017), an HPV vaccine was first introduced in 2006 and has demonstrated its effectiveness based on post-market surveillance data. Studies showed that HPV vaccines contributed to a 64% reduction in vaccine-type HPV infections among teenage girls in the U.S. (Centers for Disease Control and Prevention (CDC), 2016c) and potentially prevents 31,200 (90%) HPV related cancers per year (Centers for Disease Control and Prevention (CDC), 2017b). According to CDC guidelines, HPV vaccines can be given to people beginning at age 9 years and is recommended for ages 11 through 26 (Centers for Disease Control and Prevention (CDC), 2016b). Ideally people should receive the HPV vaccines before they become sexually active and are exposed to HPV (Centers for Disease Control and Prevention (CDC), 2016b). In 2018 the U.S. Food and Drug Administration (FDA) expanded the approved use of the HPV vaccine to include women and men aged 27 through 45 years (Food and Drug Administration (FDA), 2018). With the increase in HPV vaccination coverage (Centers for Disease Control and Prevention (CDC), 2018; Curtis et al., 2013), pharmacovigilance should be further strengthened.

The distribution and cause of adverse events (AEs) has been an integral topic of pharmacovigilance (Du et al., 2017; Harpaz et al., 2011; Tao et al., 2015; Wilson et al., 2018). Post market surveillance is one of the common approaches to investigate vaccine AEs (McPhillips & Marcuse, 2001). The Vaccine Adverse Event Reporting System (VAERS), a national early warning system that detects potential safety concerns in U.S. licensed vaccines (Vaccine Adverse Event Reporting System (VAERS), 2018), is one of the popular resources explored by various post-market vaccine safety studies (Haber et al., 2016; Iskander, Haber & Herrera, 2005; Moro et al., 2015; Slade et al., 2009; Xie et al., 2016). The topics of these studies ranged from validating results of clinical research (Block et al., 2010; Gold, Buttery & McIntyre, 2010; Li et al., 2016; Stokley et al., 2014), general description of AE types (Levi et al., 2013), identifying safety signals and causal relationships (Debeer et al., 2008; Gee et al., 2011, 2016; Lu et al., 2011; Pomfret, Gagnon & Gilchrist, 2011), AEs and differences in categories of AE across races (Huang et al., 2018). However, none of these studies investigated the association between the variation in HPV vaccination related AE categories and age or gender. In addition, the correlation or clustering among AE categories is underexplored, though it has been studied using a data source other than VAERS (Chandler et al., 2017).

This article reports on a retrospective observational study aimed to address the major two knowledge gaps above. In addition to identify differences among AEs, the novelty of this study was that it explored the underlying relationship among AEs, which could potentially advance future investigation into the biological or clinical relevance of these relationships. The investigation was based on the HPV vaccine AE records extracted from the VAERS database between 2006 and 2017.

## Materials and Methods

### Methods overview

We implemented a retrospective observational study based on the VAERS data to address the following research questions: (1) Are the frequencies of AEs different across different gender and age groups? If significant differences are observed, it is essential to develop a more precise vaccine information statement for these targeted subgroups. (2) Are there any correlations among the AEs? Specifically, we explored whether some AEs were more likely to occur together. This study included subjects aged between 9 and 25 years old who received the following vaccine types: HPVX/HPV2/HPV4/HPV9 (HPV vaccine with no brand name/HPV Cervarix/HPV Gardasil/HPV Gardasil 9). We excluded records with any missing information regarding age, gender or symptoms. The final eligible sample size was 26,023 including 21,647 females and 4,376 males.

### Data source mapping symptoms in VAERS

**Figure 1:**
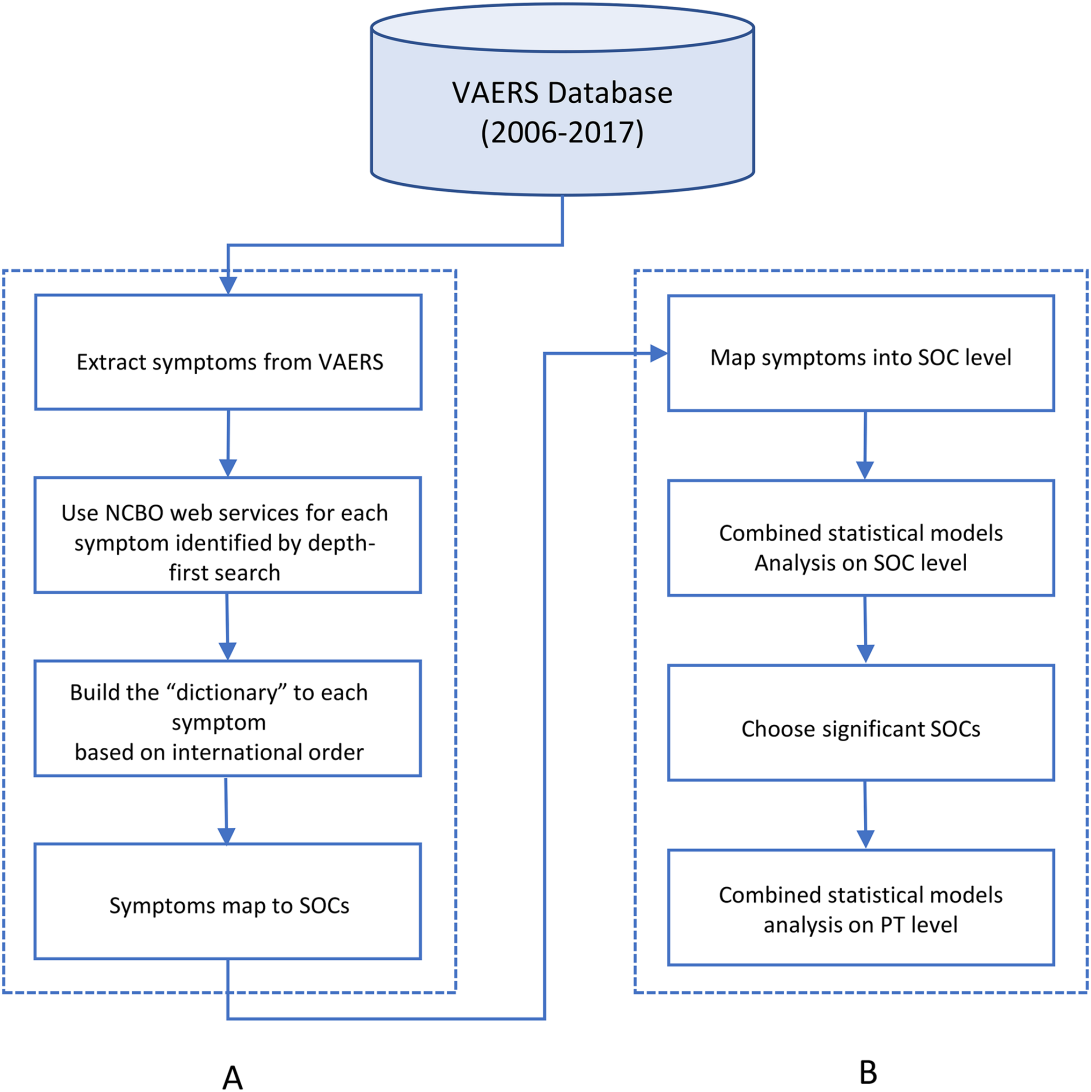
The flowchart of project. (A) was to map AEs from MedDRA PT level to MedDRA SOC level. (B) was to apply statistical models on mapped AEs.

Vaccine Adverse Event Reporting System database included nearly 47,000 reports associated with a HPV vaccine as of December 2017. AE symptoms were annotated following the guidelines of Medical Dictionary for Regulatory Activities (MedDRA) (Food and Drug Administration (FDA), 2016). MedDRA is a standardized medical terminology for medical products. MedDRA consists of a five-level structural hierarchy: system organ classes (SOC), high level group term, high level term, prefer term (PT), lowest level term (LLT) (International Council for Harmonisation, 2018). The AE symptoms in the VAERS dataset were annotated using the PT level terms. To map PT level AE to SOC level, we followed the method from a previous study and developed a dictionary-like lookup table by using the internationally agreed upon order of the SOC list (Table 1) (Du et al., 2017). The sort of the list is based upon the relative importance of each SOC, which was determined by the Expert Working Group (Medical Dictionary for Regulatory Activities (MedDRA), 2016). The “dictionary” converts the symptoms at the PT level in VAERS to a corresponding unique SOC. Based on the data update, the “dictionary” has been expanded and refined in a new round (Fig. 1).

**Table 1:** The SOC list. The MedDRA Terminology SOC List—Internationally Agreed Order. MedDRA Management Board endorsed the creation of an additional 27th SOC category named “product issue” in 2016. It has no major impact on this research.

No.	SOC
1	Infections and infestations
2	Neoplasms benign, malignant and unspecified (including cysts and polyps)
3	Blood and lymphatic system disorders
4	Immune system disorders
5	Endocrine disorders
6	Metabolism and nutrition disorders
7	Psychiatric disorders
8	Nervous system disorders
9	Eye disorders
10	Ear and labyrinth disorders
11	Cardiac disorders
12	Vascular disorders
13	Respiratory, thoracic and mediastinal disorders
14	Gastrointestinal disorders
15	Hepatobiliary disorders
16	Skin and subcutaneous tissue disorders
17	Musculoskeletal and connective tissue disorders
18	Renal and urinary disorders
19	Pregnancy, puerperium and perinatal conditions
20	Reproductive system and breast disorders
21	Congenital, familial and genetic disorders
22	General disorders and administration site conditions
23	Investigations
24	Injury, poisoning and procedural complications
25	Surgical and medical procedures
26	Social circumstances
27*	Product issues

There are 27 SOC categories defined by MedDRA. However, since the “product issue” category does not describe AEs in humans, any AE that belonged to this category was excluded from the analyses. Therefore, 26 SOCs was included in this study.

### Statistical methods

Our analyses consisted of two major inter-coherent parts based on the retrospective cohort study design. The first part was to address the hypothesis whether individual SOC level AEs contributed to the total AE reports differently, and whether age and gender profiles vary across these individual SOC AEs. In addition to investigate the difference, in the second part of the analyses we explored the hypothesis that correlations or clustering patterns might exist among some AE categories, and examined the lower AEs term (PT level) that contributed to these potential observed correlations.

Corresponding to the analyses scheme part one, an independent *t*-test was performed to assess the age distribution across gender and specific SOCs. A multivariate logistic regression model was developed to evaluate the risk of developing a specific SOC, accounting for potential confounding effects by age and gender. Odds ratio (OR) greater than 1 indicates an increased odd of getting a specific SOC, whilst a less than 1 OR suggests decreased odds. A zero truncated Poisson regression model was used to model the number of events because patients need to have at least one AE in order to be captured by the VAERS system. Zero truncated Poisson regression model was performed to assess the number of individual SOC cases per year adjusted for gender and age.

For any SOCs that were statistically significant from either the logistic regression model or zero truncated Poisson regression model, the lower-level symptoms (PT) attributed to these SOCs were further investigated using the multivariate logistic regression model adjusted for age and gender.

Finally, corresponding to the analyses scheme part two, pairwise Pearson correlation and un-supervised hierarchy clustering analyses were conducted to explore the interrelationships and clustering patterns across AEs. The analyses were first based on SOC level to identify the general pattern of the high-level AEs. Then PT level clustering analyses were performed on the SOC level AE that contributed to the majority of the AE cases and its most correlated three SOC level AEs. The hierarchical clustering method is based on the Euclidean distance between the vectors of Pearson correlation using a complete linkage algorithm. The optimal number of clusters was determined by the average silhouette method (Rousseeuw, 1987). Correlation network figures were then developed to summarize findings regarding correlation strength and clustering patterns of the PT level AEs. Only those with statistically significantly weak or above correlation (*r* > 0.2) are included in the Figures. Statistical significance level was defined as adjusted *p*-value < 0.05, after accounting for multiple testing using the Holm method (Aickin & Gensler, 1996). The details of the Holm method have been discussed elsewhere, in general, it controls type-I error (false positive) in a less conservative way than Bonferroni correction (Bland & Altman, 1995) with lowered risks in type II error (false negative).

All statistical analyses were based on R version 3.5.1 (R Core Team, 2018).

## Results

Table 2 lists the age distribution of SOCs by gender evaluated through independent *t*-test. Overall, the first vaccination age of females was relatively older than males according to the VAERS data (16.318 vs 14.14, 95% CI [2.073–2.283]), the difference was statistically significant (*p* < 0.0019). Similar distribution was observed across all individual SOC subgroups. The age difference was statistically significant among all SOCs except for neoplasms benign, malignant and unspecified (including cysts and polyps), ear and labyrinth disorders, hepatobiliary disorders and social circumstances.

**Table 2:** Age distribution of SOCs by gender, VAERS 2006–2017*.

SOC	Female mean age	Male mean age	Mean difference (95% CI)		*p*-value**	Sample size
ALL	16.318	14.14	2.073	2.283	<0.001	26,023
Nervous system disorders	15.853	14.506	1.202	1.492	<0.001	12,369
General disorders and administration site conditions	16.327	13.762	2.402	2.727	<0.001	10,537
Skin and subcutaneous tissue disorders	15.935	13.342	2.4	2.785	<0.001	6,688
Investigations	16.792	14.991	1.519	2.08	<0.001	5,790
Gastrointestinal disorders	16.277	14.248	1.773	2.284	<0.001	4,706
Musculoskeletal and connective tissue disorders	16.554	14.769	1.471	2.098	<0.001	4,241
Immune system disorders	16.169	14.144	1.742	2.307	<0.001	3,802
Injury, poisoning and procedural complications	15.941	14.74	0.91	1.49	<0.001	2,791
Psychiatric disorders	15.846	14.301	1.227	1.864	<0.001	2,711
Vascular disorders	15.776	14.243	1.229	1.837	<0.001	2,650
Infections and infestations	16.793	14.347	1.868	3.022	<0.001	1,962
Cardiac disorders	16.497	14.376	1.694	2.547	<0.001	1,803
Metabolism and nutrition disorders	16.113	13.331	2.245	3.32	<0.001	1,178
Respiratory, thoracic and mediastinal disorders	15.913	14.113	1.276	2.324	<0.001	1,083
Surgical and medical procedures	17.42	14.8	1.95	3.286	<0.001	979
Blood and lymphatic system disorders	16.779	15.176	0.782	2.423	0.002	773
Social circumstances	16.181	15.173	0.072	1.945	0.106	625
Eye disorders	15.511	14.505	0.281	1.731	0.034	621
Reproductive system and breast disorders	17.574	14.357	1.661	4.772	0.004	565
Endocrine disorders	16.55	14.5	0.751	3.35	0.032	537
Ear and labyrinth disorders	16.069	15.808	−1.381	1.904	0.847	228
Neoplasms benign, malignant and unspecified	16.769	13.583	0.873	5.499	0.044	220
Renal and urinary disorders	16.324	13.882	1.037	3.847	0.011	205
Hepatobiliary disorders	16.917	16.2	−1.182	2.615	0.847	53

**Notes:**

* SOCs congenital, familial and genetic disorders and pregnancy, puerperium and perinatal conditions were not included because their cases were exclusively females.

** *p*-value was adjusted for multiple testing using Holm method.

Table 3 illustrates the association between the rate of developing any SOCs across gender and age. The interaction term between gender and age from the pooled model was statistically significant (*p* < 0.0019), which indicated the effect of age on developing any SOCs varied by gender. The subsequent stratification analyses shed further light on the difference of the effect by age variable. As shown in the male-only Poisson Regression model, with 1-year increase in age, the rate of developing SOCs among the observed cohort increased by 0.01 cases per person-year. In contrast, in the female-only model, each 1-year increase in age was associated with 0.004 decrease case per person-year on average.

**Table 3:** Zero truncated Poisson regression of total population and by gender. The association between the rate of developing any SOCs across gender and age.

		Coefficient	*p*-value
Pooled	Age (Years)	−0.004	<0.001
	Male (Ref: Female)	−0.482	<0.001
	Age (Years) * Male	0.014	<0.001
Male	Age (Years)	0.010	<0.001
Female	Age (Years)	−0.004	<0.001

Table 4 presents the odds of developing specific SOCs adjusting for gender, age and interaction term between gender and age. This table only includes top 10 SOCs with which contributed to the most AE. A complete table of 24 SOCs can be found in Table S1. Using nervous system disorders as an example for the interpretation of effects of the three variables: Among females, each 1 year increase in age is associated with 0.055 (1 − exp(−0.057 × 1 + 0.131 × 1 × 0)) decrease in the odds of developing nervous system disorders. In contrast, among males, each 1 year increase in age is associated with (exp(−0.057 × 1 + 0.131 × 1 × 1) − 1) 0.077 increased odds of developing nervous system disorders.

**Table 4:** Logistic Regression by Individual SOCs adjusted for gender, age and interaction term between gender and age. The odds of developing specific SOCs adjusting for gender, age and interaction term between gender and age. This table only includes top 10 SOCs with which contributed to the most AE. A complete table of 24 SOCs can be found in Table S1.

No.			Coefficient	Standard error	*p*-value
4	Immune system disorders	Age (in Year)	−0.011	0.005	0.225
		Gender (Male:1, Female:0)	−0.35	0.227	1.000
		Age * Gender	0.012	0.015	1.000
7	Psychiatric disorders	Age (in Year)	−0.034	0.006	<0.001
		Gender (Male:1, Female:0)	−0.951	0.259	0.004
		Age * Gender	0.053	0.018	0.043
8	Nervous system disorders	Age (in Year)	−0.057	0.003	<0.001
		Gender (Male:1, Female:0)	−2.018	0.157	<0.001
		Age * Gender	0.131	0.011	<0.001
12	Vascular disorders	Age (in Year)	−0.039	0.006	<0.001
		Gender (Male:1, Female:0)	−0.737	0.249	0.043
		Age * Gender	0.052	0.017	0.039
14	Gastrointestinal disorders	Age (in Year)	−0.003	0.004	1.000
		Gender (Male:1, Female:0)	−0.534	0.213	0.148
		Age * Gender	0.017	0.014	1.000
16	Skin and subcutaneous tissue disorders	Age (in Year)	−0.033	0.004	<0.001
		Gender (Male:1, Female:0)	1.725	0.181	<0.001
		Age * Gender	−0.106	0.013	<0.001
17	Musculoskeletal and connective tissue disorders	Age (in Year)	0.018	0.004	0.001
		Gender (Male:1, Female:0)	−1.134	0.226	<0.001
		Age * Gender	0.054	0.015	0.006
22	General disorders and administration site conditions	Age (in Year)	0.001	0.003	1.000
		Gender (Male:1, Female:0)	1.236	0.159	<0.001
		Age * Gender	−0.075	0.011	<0.001
23	Investigations	Age (in Year)	0.039	0.004	<0.001
		Gender (Male:1, Female:0)	−1.465	0.215	<0.001
		Age * Gender	0.058	0.014	0.001
24	Injury, poisoning and procedural complications	Age (in Year)	−0.027	0.006	<0.001
		Gender (Male:1, Female:0)	−1.011	0.22	<0.001
		Age * Gender	0.097	0.014	<0.001

As for PT level analyses, nervous system disorders was targeted because it contributed to the most adverse events (12,378) in VAERS data. As shown in Table 5, a disproportional reporting of PT level terms was found between the genders. For example, males experienced fewer headaches and hypoesthesia than females, 19.40% vs 24.20% and 3.97% vs 7.9%, respectively.

**Table 5:** Chi-square test of PT level symptoms from nervous system disorders*.

		Female		Male		*p*-value
		*N*	%	*N*	%	
Dizziness	No	6,982	67.50	1,359	66.70	0.454
	Yes	3,357	32.50	680	33.30	
Syncope	No	7,348	71.10	1,470	72.10	0.365
	Yes	2,991	28.90	569	27.90	
Headache	No	7,838	75.80	1,644	80.60	<0.001
	Yes	2,501	24.20	395	19.40	
Loss of consciousness	No	8,866	85.75	1,716	84.16	0.067
	Yes	1,473	14.25	323	15.84	
Hypoaesthesia	No	9,522	92.10	1,958	96.03	<0.001
	Yes	817	7.90	81	3.97	

**Note:**

* Only top five PT-level terms were demonstrated.

**Figure 2:**
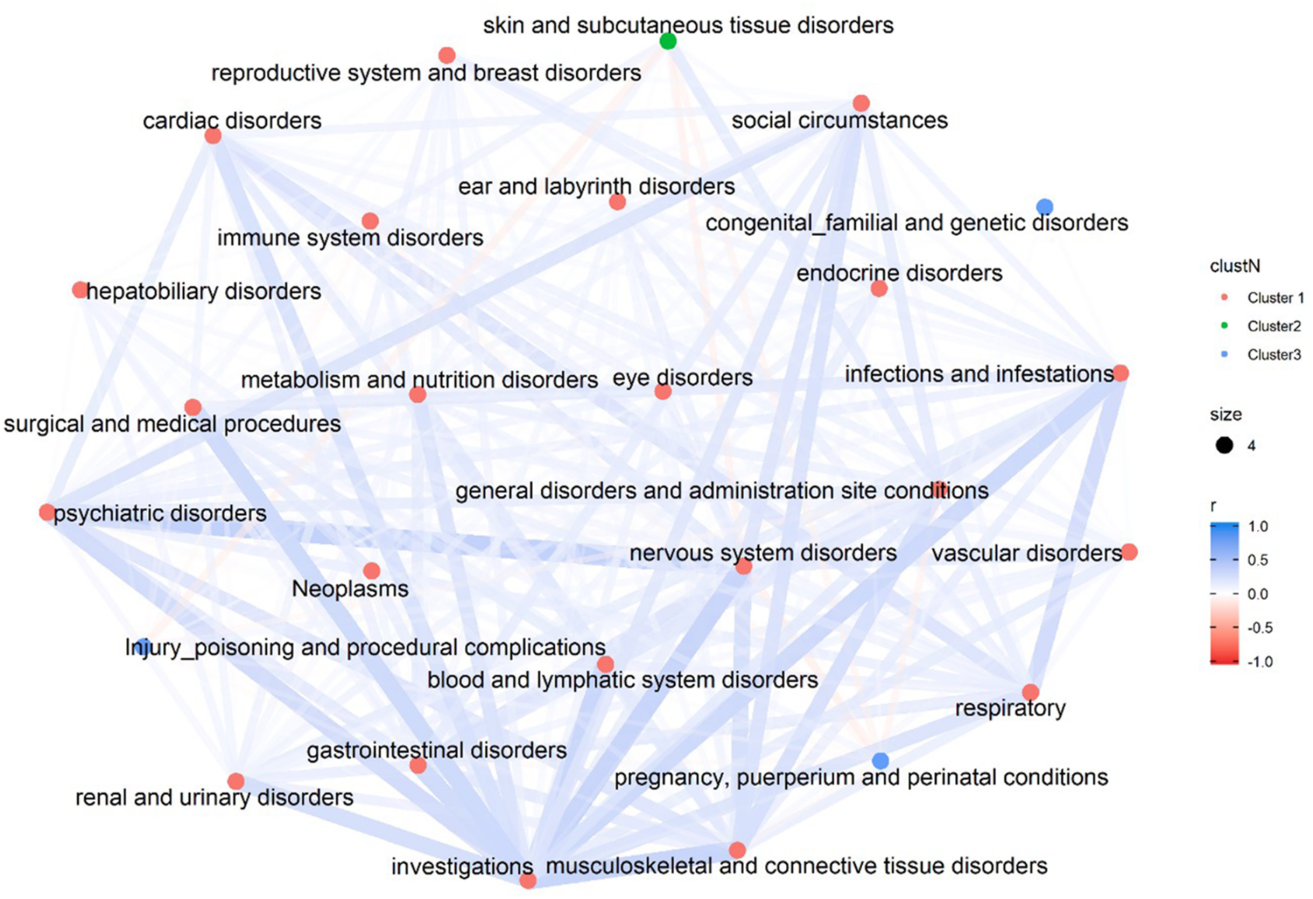
Correlation Network of 26 SOCs. The overall correlation among the 26 SOCs. The colors of the nodes differentiate the clusters of each SOC, which is determined by the hierarchical clustering analyses using a complete linkage algorithm.

Figure 2 illustrates the overall correlation among the 26 SOCs. The majority of the SOCs seem to be positively connected except five pairs which demonstrate weak negative correlation (skin and subcutaneous tissue disorders + injury poisoning and procedural complications; skin and subcutaneous tissue disorders + nervous system disorders; skin and subcutaneous tissue disorders + pregnancy, puerperium and perinatal conditions; nervous system disorders + pregnancy, puerperium and perinatal conditions; pregnancy, puerperium and perinatal conditions + general disorders and administration site conditions).

**Figure 3:**
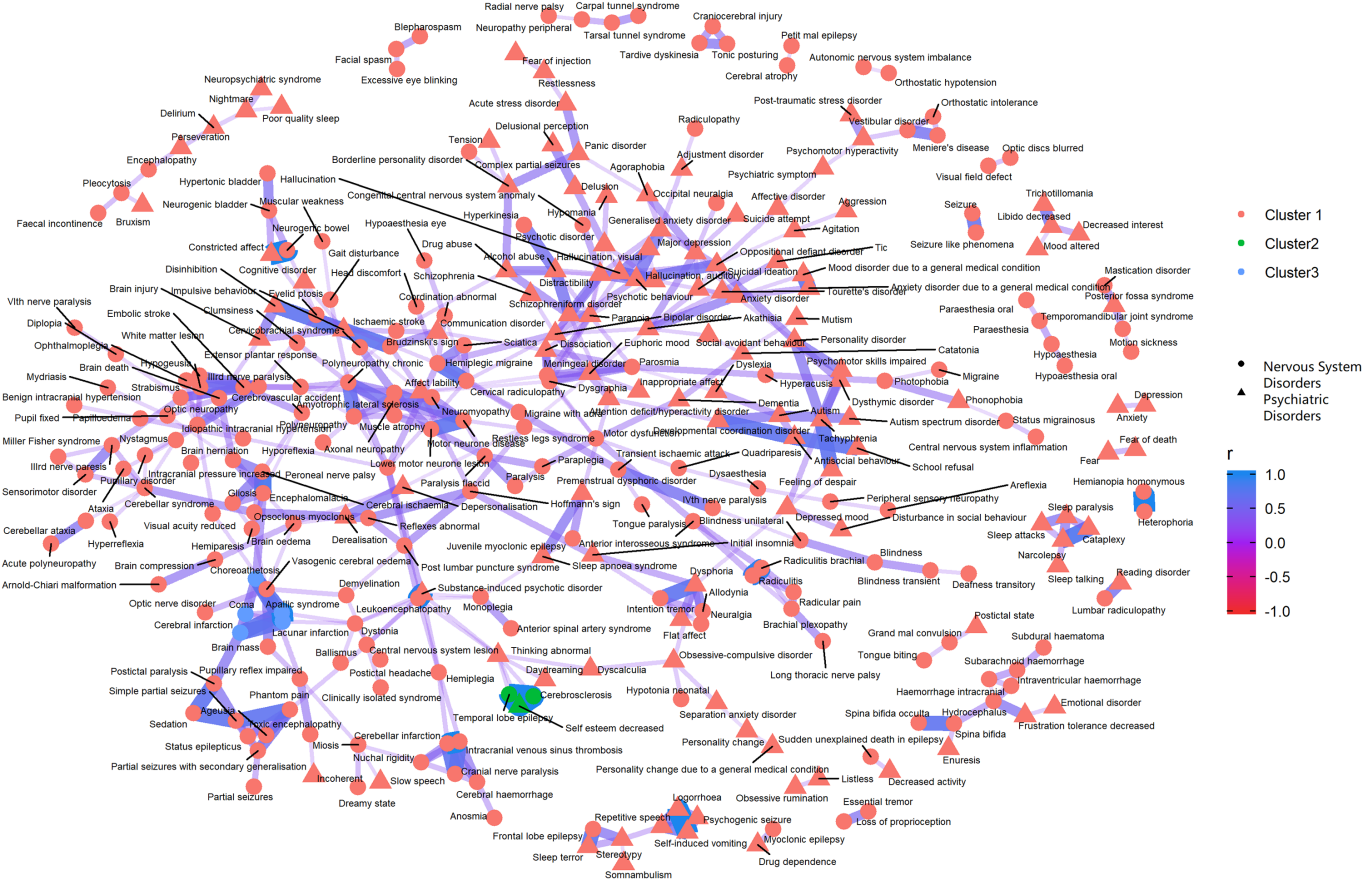
Correlation network of pairwise Pearson correlation of PT-term level AEs from nervous system disorders and psychiatric disorders. The PT-level interrelationship among AEs from nervous system disorders and psychiatric disorders.

**Figure 4:**
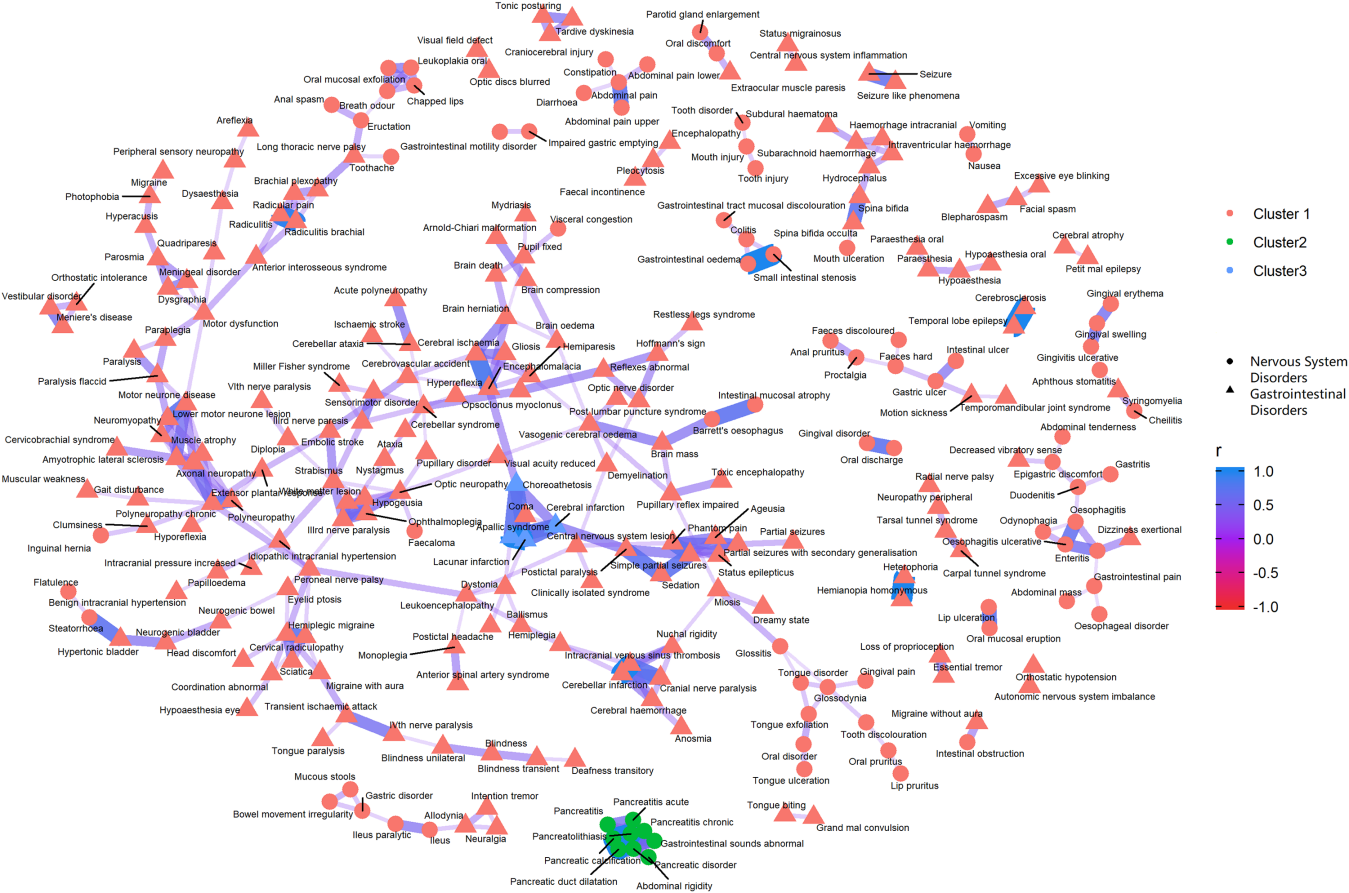
Correlation network of pairwise Pearson correlation of PT-term level AEs from nervous system disorders and gastrointestinal disorders. The PT-level interrelationship among AEs from nervous system disorders and gastrointestinal disorders.

**Figure 5:**
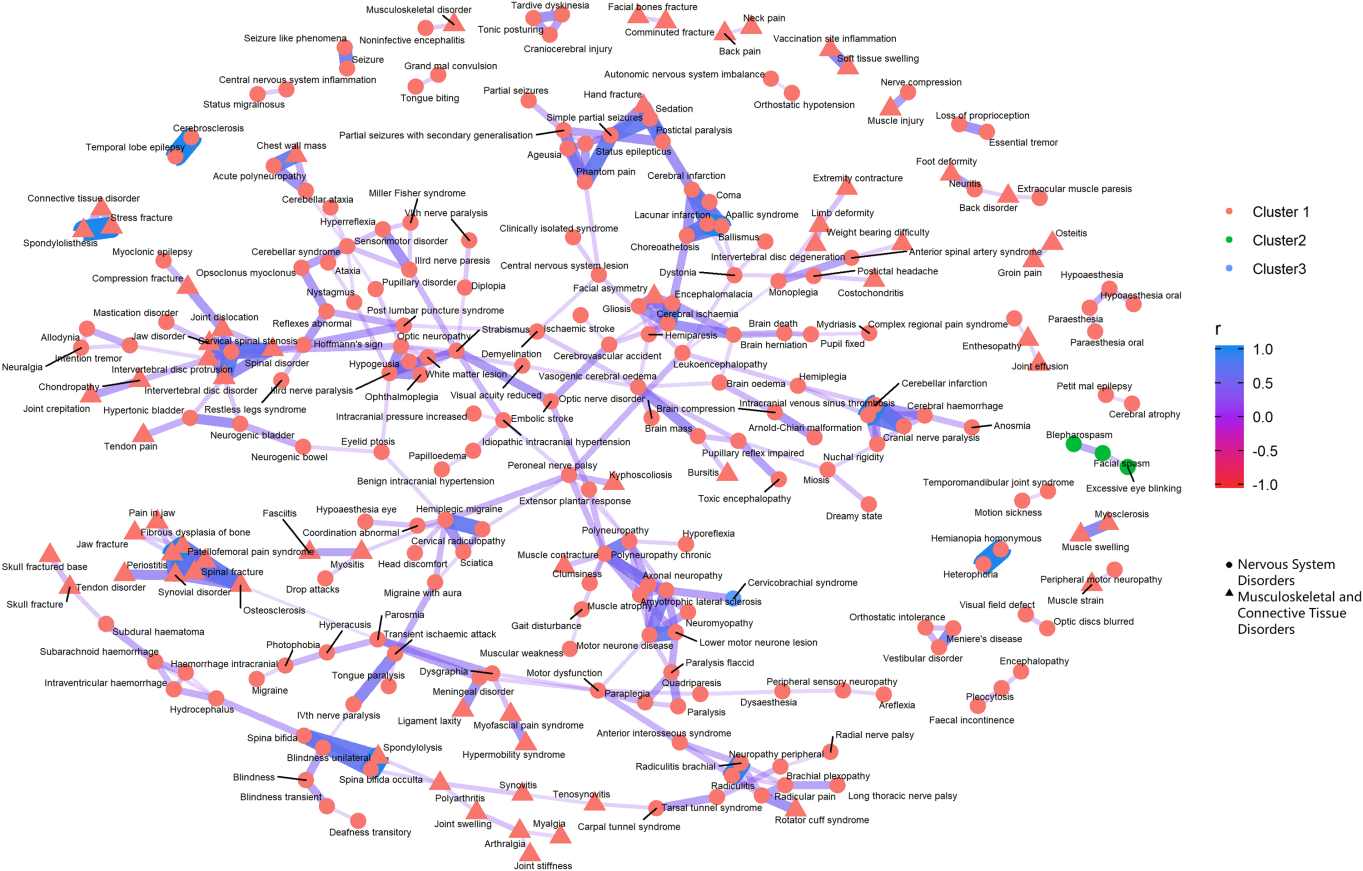
Correlation network of pairwise Pearson correlation of PT-term level AEs from nervous system disorders and musculoskeletal and connective tissue disorders. The PT-level interrelationship among AEs from nervous system disorders and musculoskeletal and connective tissue disorders.

Figures 3–5 show the PT-level interrelationship among AEs from four SOC groups: nervous system disorders, psychiatric disorders, gastrointestinal disorders and musculoskeletal and connective tissue disorders. The first one contributed the most AEs while the other three demonstrate clinical relevance and strongest correlation with nervous systems disorders (*r* = 0.357, 0.215, 0.261, respectively). The figures only display those AEs with Pearson correlation *r* > 0.2, and adjusted *p*-value < 0.05 (Holm method). An inter-SOCs correlation of the PTs was observed among nervous system disorders + psychiatric disorders and nervous system disorders + musculoskeletal and connective tissue disorders; the relatively strong correlations were not limited to PTs attributed to the same SOC category. For example, the correlation between Photophobia (nervous system disorders) and phonophobia (psychiatric disorders) was 0.38 (*p* < 0.05). This frequently observed inter-SOCs correlation may be due to the close clinical relationship among these three SOCs. However, this pattern was much less common in pairs of nervous system disorders and gastrointestinal disorders. Most of the weak or above correlations (*r* > 0.2) were found among PTs from the same SOC category. Only a few inter-SOCs correlation were identified, such as the pair of Intestinal obstruction and Migraine without aura (*r* = 0.41).

As for the clustering analyses, most of the AEs were recommended to group into one large cluster, with the rest categorized into two small clusters. The PTs within the same cluster that demonstrated the most distinct clinical relevance were pancreatitis related AEs, abdominal rigidity, and gastrointestinal sounds abnormal (green cluster, Fig. 4).

## Discussion

Our analyses revealed a variation in the distribution of AEs across gender and age groups. Generally, males reported AEs at younger first vaccination ages than females. None of the SOC level AEs which demonstrated statistical significance with age and gender met FDA's standards of serious AEs. On average, each person experienced four SOC level AEs. Among males, older vaccination age was associated with a slight increase in reported SOC level AEs, while the trend was reversed among females.

In the clustering analyses which investigated the top SOC subgroup nervous system disorders and its three most correlated AE subgroups psychiatric disorders, musculoskeletal and connective tissue disorders and gastrointestinal disorders, we identified many weak and above (*r* > 0.2) inter subgroup correlation among PT pairs from nervous system disorders/psychiatric disorders and nervous system disorders/musculoskeletal and connective tissue disorders. However, the same level correlations among the pool of AEs by nervous system disorders and gastrointestinal disorders were mostly confined to PTs from the same SOC category. While the silhouette recommended that PT level AEs be divided into three clusters, whether such categorization is clinically informative demands further investigations.

The results of all of the statistical analyses from this study should be interpreted with caution. Since VAERS exclusively collected adverse reactions data (events post vaccination), the model based on its data only evaluates the risk or rate of developing specific adverse reactions among those who have already experienced adverse reactions. Our findings do not imply any real risk of developing adverse events after receiving HPV vaccination in the general population. Based on the CDC data, roughly half of adolescents received HPV vaccination by 2017 (Centers for Disease Control and Prevention (CDC), 2017a). Considering the 42 million population among the 10–19 years old group (The Statista Portal, 2017), we estimate the number of vaccinated adolescents to be approximately 21 million. This number should be even larger if considering the cohort effect (those who entered the adult cohort before 2017). However, only 26,023 adverse events were reported between 2006 and 2017 to VAERS, among which the non-serious AEs (dizziness, syncope, headache, etc. Table 4) contributed to the majority. In addition, events such as Syncope, fainting and short-term loss of consciousness are common in adolescents regardless of the vaccination type. Therefore, our study didn't reveal any findings that contradict the current HPV vaccine safety profile.

The clustering pattern of the PT level terms was determined using the un-supervised learning methodology instead of clinical interpretations. Further studies are warranted to explore the biological pathways between the correlated adverse reactions. Due to the limitation as a passive surveillance system, issues such as under-reporting, over-reporting, inconsistent reporting quality etc. exist (Moro et al., 2015). Especially, VAERS accepts all reports without judging whether the event was caused by the vaccine (Vaccine Adverse Event Reporting System (VAERS), 2019). The observed statistical significance in the analyses does not imply cause-effect relationships and needs to be further validated in relevant clinical studies.

## Conclusions

To the best of our knowledge, this is the first investigation that systematically reviewed post-market safety profiles of HPV vaccines using VAERS data. The analyses revealed a different distribution pattern of AEs among different gender and age subgroups. This research contributes to the notion that the benefits of vaccination outweigh the potential risks.

We found 13 SOCs with significant differences in first vaccination. Based on the clustering analyses of selected AEs from nervous system disorders, psychiatric disorders, musculoskeletal and connective tissue disorders and gastrointestinal disorders, we identified inter-dependences among AEs from different SOC level categories. VAERS data provide valuable HPV vaccines safety data. However, findings from this study should be interpreted with caution. Further clinical studies are warranted to understand the heterogeneity of AEs reporting across subgroups and the biology pathways among the statistically correlated AEs.

## Supplemental Information

10.7717/peerj.7490/supp-1 Supplemental Information 1 Logistic Regression by all SOCs adjusted for Gender, Age and Interaction Term Between Gender and Age. The odds of 24 SOCs adjusting for gender, age and interaction term between gender and age.

## Additional Information and Declarations

### Data Availability

The following information was supplied regarding data availability: Raw data is available in Table 4 and Table S1. Table 4 presents the odds of developing specific SOCs adjusting for gender, age and interaction term between gender and age. This table only includes SOCs with statistical significance in either age or gender parameters. A complete table of 26 SOCs can be found in Table S1.
