# Supplementary material for: Investigating safety profiles of human papillomavirus vaccine across group differences using VAERS data and MedDRA
Source: PeerJ. 2019 Aug 20;7:e7490. doi: 10.7717/peerj.7490 (PMC6707342; doi:10.7717/peerj.7490)
Supplement: Supplemental Information 1 — The odds of 24 SOCs adjusting for gender, age and interaction term between gender and age. [file peerj-07-7490-s001.docx]

**Table 4. Logistic Regression by Individual SOCs adjusted for Gender, Age and Interaction Term Between Gender and Age**

| **No.** |  |  | **Coefficient** | | | **Standard Error** | | **P-value** | |
| --- | --- | --- | --- | --- | --- | --- | --- | --- | --- |
| 1 | infections and infestations | Age (in Year) | | 0.032 | 0.006 | | <0.001 | |  |
|  |  | Gender(Male:1,Female:0) | | -0.468 | 0.361 | | 1.000 | |  |
|  |  | Age*Gender | | -0.009 | 0.024 | | 1.000 | |  |
| 2 | Neoplasms | Age (in Year) | | 0.028 | 0.017 | | 0.807 | |  |
|  |  | Gender(Male:1,Female:0) | | 0.108 | 1.469 | | 1.000 | |  |
|  |  | Age*Gender | | -0.093 | 0.105 | | 1.000 | |  |
| 3 | blood and lymphatic system disorders | Age (in Year) | | 0.029 | 0.01 | | 0.025 | |  |
|  |  | Gender(Male:1,Female:0) | | -1.464 | 0.517 | | 0.060 | |  |
|  |  | Age*Gender | | 0.069 | 0.033 | | 0.421 | |  |
| 4 | immune system disorders | Age (in Year) | | -0.011 | 0.005 | | 0.225 | |  |
|  |  | Gender(Male:1,Female:0) | | -0.35 | 0.227 | | 1.000 | |  |
|  |  | Age*Gender | | 0.012 | 0.015 | | 1.000 | |  |
| 5 | endocrine disorders | Age (in Year) | | 0.015 | 0.011 | | 1.000 | |  |
|  |  | Gender(Male:1,Female:0) | | -2.668 | 1.47 | | 0.765 | |  |
|  |  | Age*Gender | | 0.022 | 0.099 | | 1.000 | |  |
| 6 | metabolism and nutrition disorders | Age (in Year) | | -0.014 | 0.008 | | 0.794 | |  |
|  |  | Gender(Male:1,Female:0) | | 0.699 | 0.472 | | 1.000 | |  |
|  |  | Age*Gender | | -0.089 | 0.034 | | 0.135 | |  |
| 7 | psychiatric disorders | Age (in Year) | | -0.034 | 0.006 | | <0.001 | |  |
|  |  | Gender(Male:1,Female:0) | | -0.951 | 0.259 | | 0.004 | |  |
|  |  | Age*Gender | | 0.053 | 0.018 | | 0.043 | |  |
| 8 | nervous system disorders | Age (in Year) | | -0.057 | 0.003 | | <0.001 | |  |
|  |  | Gender(Male:1,Female:0) | | -2.018 | 0.157 | | <0.001 | |  |
|  |  | Age*Gender | | 0.131 | 0.011 | | <0.001 | |  |
| 9 | eye disorders | Age (in Year) | | -0.055 | 0.012 | | <0.001 | |  |
|  |  | Gender(Male:1,Female:0) | | -1.563 | 0.518 | | 0.039 | |  |
|  |  | Age*Gender | | 0.093 | 0.035 | | 0.106 | |  |
| 10 | ear and labyrinth disorders | Age (in Year) | | -0.016 | 0.018 | | 1.000 | |  |
|  |  | Gender(Male:1,Female:0) | | -2.896 | 0.913 | | 0.024 | |  |
|  |  | Age*Gender | | 0.162 | 0.056 | | 0.062 | |  |
| 11 | cardiac disorders | Age (in Year) | | 0.012 | 0.007 | | 0.687 | |  |
|  |  | Gender(Male:1,Female:0) | | -0.317 | 0.312 | | 1.000 | |  |
|  |  | Age*Gender | | 0.014 | 0.021 | | 1.000 | |  |
| 12 | vascular disorders | Age (in Year) | | -0.039 | 0.006 | | <0.001 | |  |
|  |  | Gender(Male:1,Female:0) | | -0.737 | 0.249 | | 0.043 | |  |
|  |  | Age*Gender | | 0.052 | 0.017 | | 0.039 | |  |
| 13 | respiratory, thoracic and mediastinal disorders | Age (in Year) | | -0.027 | 0.009 | | 0.019 | |  |
|  |  | Gender(Male:1,Female:0) | | -0.639 | 0.418 | | 1.000 | |  |
|  |  | Age*Gender | | 0.025 | 0.029 | | 1.000 | |  |
| 14 | gastrointestinal disorders | Age (in Year) | | -0.003 | 0.004 | | 1.000 | |  |
|  |  | Gender(Male:1,Female:0) | | -0.534 | 0.213 | | 0.148 | |  |
|  |  | Age*Gender | | 0.017 | 0.014 | | 1.000 | |  |
| 15 | hepatobiliary disorders | Age (in Year) | | 0.037 | 0.035 | | 1.000 | |  |
|  |  | Gender(Male:1,Female:0) | | -2.662 | 2.041 | | 1.000 | |  |
|  |  | Age*Gender | | 0.136 | 0.122 | | 1.000 | |  |
| 16 | skin and subcutaneous tissue disorders | Age (in Year) | | -0.033 | 0.004 | | <0.001 | |  |
|  |  | Gender(Male:1,Female:0) | | 1.725 | 0.181 | | <0.001 | |  |
|  |  | Age*Gender | | -0.106 | 0.013 | | <0.001 | |  |
| 17 | musculoskeletal and connective tissue disorders | Age (in Year) | | 0.018 | 0.004 | | <0.001 | |  |
|  |  | Gender(Male:1,Female:0) | | -1.134 | 0.226 | | <0.001 | |  |
|  |  | Age*Gender | | 0.054 | 0.015 | | 0.006 | |  |
| 18 | renal and urinary disorders | Age (in Year) | | 0.0004 | 0.018 | | 1.000 | |  |
|  |  | Gender(Male:1,Female:0) | | -0.4 | 1.214 | | 1.000 | |  |
|  |  | Age*Gender | | -0.029 | 0.085 | | 1.000 | |  |
| 20 | reproductive system and breast disorders | Age (in Year) | | 0.077 | 0.01 | | <0.001 | |  |
|  |  | Gender(Male:1,Female:0) | | -1.118 | 1.259 | | 1.000 | |  |
|  |  | Age*Gender | | -0.054 | 0.085 | | 1.000 | |  |
| 22 | general disorders and administration site conditions | Age (in Year) | | 0.001 | 0.003 | | 1.000 | |  |
|  |  | Gender(Male:1,Female:0) | | 1.236 | 0.159 | | <0.001 | |  |
|  |  | Age*Gender | | -0.075 | 0.011 | | <0.001 | |  |
| 23 | investigations | Age (in Year) | | 0.039 | 0.004 | | <0.001 | |  |
|  |  | Gender(Male:1,Female:0) | | -1.465 | 0.215 | | <0.001 | |  |
|  |  | Age*Gender | | 0.058 | 0.014 | | 0.001 | |  |
| 24 | injury, poisoning and procedural complications | Age (in Year) | | -0.027 | 0.006 | | <0.001 | |  |
|  |  | Gender(Male:1,Female:0) | | -1.011 | 0.22 | | <0.001 | |  |
|  |  | Age*Gender | | 0.097 | 0.014 | | <0.001 | |  |
| 25 | surgical and medical procedures | Age (in Year) | | 0.069 | 0.008 | | <0.001 | |  |
|  |  | Gender(Male:1,Female:0) | | -0.433 | 0.491 | | 1.000 | |  |
|  |  | Age*Gender | | -0.003 | 0.032 | | 1.000 | |  |
| 26 | social circumstances | Age (in Year) | | -0.009 | 0.011 | | 1.000 | |  |
|  |  | Gender(Male:1,Female:0) | | -2.387 | 0.648 | | 0.004 | |  |
|  |  | Age*Gender | | 0.106 | 0.041 | | 0.135 | |  |
